# Annotations

**Published:** 1890-12-20

**Authors:** 


### Annotations.

"An Old Song."

"So likewise we honour the mighty immortals
As if they were men, and did on a grand scale
What good men on small scale do or fain would."

Thus Goethe! It is not good for science, any more than for man, to be alone; it needs to be married to kindly thought and noble deed. The man of science will listen to Goethe; will he listen to One who spoke before Goethe, and in a much cruder time; One to whom Goethe very reverently listened? There can be no check to scientific progress in loving our neighbours as ourselves. If we were in poverty or in sorrow we should not think a man necessarily a "humbug" and a "hypocrite" if he gave us genuine sympathy and practical help. There is no more actual, tangible, and verifiable fact in the world than the want, suffering, and helpless distress of many of our neighbours in London and other large towns. It is a fact which remains indisputable, whatever test be applied. Keen frost makes a child hungry, and if his hunger be only half appeased it soon returns again, and becomes very like an inward, gnawing wolf. To be hungry all day and to go to bed hungry; to have no fire in the room and very few bedclothes; that is a decidedly hard fact of science to those who experience it. Charity organisation is a good thing, men of science; but just at Christmas time, when the prosperous are going to enjoy themselves, and the cold has chosen to be as keen and eager and nipping as frost and east winds can make it, charity bread and butter, charity fire, and charity blankets are a good deal better than charity organisation. We do not believe in indiscriminate giving, neither do we believe in indiscriminate withholding. The world had a little sense before modern science and charity organisation were born. It produced a Milton, and a Shakespeare, and a Christ. This is Christmas time. Let us be merely human, simply brotherly, for a few days! Take away your scrutinies and your official investigations! Even at ordinary times no man can be quite certain whether they come from heaven or another place. But in Christmas week we are certain they have a sulphurous smell. "Peace upon earth and goodwill toward men" has at least as fine a "ring" about it as any of your modern science-songs. Translate it into practice for a time. The results might conceivably be as useful and as lasting, possibly even as wonderful, as those produced by the "steamship and the railway," and the other fruits of latter day labours. Let the old song be both the moral and the scientific code for one week.

American and English Girls.

Madame Adam, the Editress of the *Nouvelle Révue*, contributes an article to the *North American Review* for October, in which she not only paints the American girl in colours so impossible that they are ridiculous, but compares her with the English girl in a way to make the latter die of envy, if she does not happen to have more sense. "The unfortunate English girls have boyish ways," according to this veracious Frenchwoman. "Their lack of elegance and their manner of walking have prejudiced them in the eyes of their French sisters." Alas, what a calamity! Now let us hear what is said of the Americans: An American girl is "enviable, and too often irresistible; she must, therefore, be imitated from afar in all that is seductive—her sincerity, her spontaneousness, her life—while the delicate charm of French education is retained. The great charm of the American girl in Europe is that she combines at once the purity of the young girl and the coquetry of the young married woman. Thus in the old world she is certain of attracting all the yonng men who have abused life, who are a little *blasé*, and who, to be captivated have need of what they call *du montant*." In all this there is as much actual truth and sense as there is food value in the sirloin of beef that is painted on the butcher's sign-board. The real fact is that the American girl is very much like any other girl; and she may be quite sure that when a Frenchwoman with a faculty for exaggerated talk makes an effort at systematic flattery, she has some object in view. Perhaps Madame Adam's sister or particular friend has a finishing school for American girls somewhere in Paris. Is it such an honour to attract "all the young men who have abused life, and are a little *blasé*"? Is it an unusual delight to ride a *blasé* horse: a miserable old screw that has been hunted four days a-week for a couple of seasons, and after that has done a year or two in a "growler" in the London streets? What the "screw" is to the six-year-old hunter who is doing his first season, that the *blasé* man is to the honest and manly fellow who has lived a clean life and followed his profession, or prepared himself for the administration of the estate that will one day be his, like a man of honour and a gentleman. Does any mortal man, or woman either, admire a *blasé* tom-cat? The *blasé* man is getting out of date, as Madame Adam would know if she were young, and as worldly-wise as she thinks she is. What religion failed to do by herself science is now helping her to accomplish. Fathers and young men are learning to understand the true value of a constitution worn out and made worse than worthless in early life. Monkeys and men without brains may still persist in making the first ten or fifteen years of adult life a period for the indulgence of the animal passions. But no one who intends to play the part of a man in the world does so. Poor Madame Adam: vapid and wordy! She probably does not even understand that the ideals she holds up are unworthy of intelligent children, much less of grown-up girls and women. If it be the fortune of American girls to attract the withered and brainless wrecks of European manhood, we can but be exceedingly sorry for them. The English girls whom this flighty Frenchwoman so ridiculously patronises may be quite content if the husbands that fall to their lot are the robust men of brains and character who rule States and do the solid work of the world. Poor American girls, if the Madame Adams of Europe are to be your guides and models! Do not forget the Parisian finishing school, which may possibly have such an intimate connection with Madame Adam's article!

An Endowment for the Bideford Hospital.

There are some circumstances connected with the Bideford Hospital, or rather Infirmary, as it is called, which deserve consideration. One of the chief is that it has fourteen beds, eleven of which are always occupied, and it treats annually more than 500 out-patients; and yet its expenditure hardly exceeds £400 a year, that is to say, the average cost per bed per annum is less than £40. That is a good record. Another important fact is that the hospital has a large honorary staff, consisting of five ordinary medical attendants and one consulting physician. A third particular that strikes the hospital critic is that the nursing staff is as sufficient as the medical: it consists of a matron and two nurses. Here, then, is a small hospital which is, what every hospital ought to be, thoroughly well equipped; and yet the annual expenditure is kept within limits of the most moderate kind. Why should we single out the Bideford Hospital and state these facts about it at the present time? Because the managers are now making an effort to improve its position; and the facts we have stated are such as to inspire public confidence in the excellence of the management, and to encourage all right-minded people to support the new departure with liberality and enthusiasm. The managers desire to raise an endowment fund for the hospital of at least £2,000. We should have been pleased if they could have seen their way to ask for five thousand instead of two. Endowments are like well-drilled standing armies in weak countries: they are excellent for defence, and assure the permanence of a good work. In September last a bazaar was held at Bideford for the hospital, at which the very handsome sum of £1,100 was cleared, after the payment of all expenses. Since that time Mr. Gloag, of Bideford, has given a donation of £100; Mrs. Brown has also given a £100, and Mr. W. L. Vellacott £20. The late Lieut.-Colonel Wheeler left a legacy of a £100, and Miss Poole a legacy of £300. These various sums amount to £1,740, leaving £260 still to be raised to complete the £2,000. It occurs to us that the managers would be made very happy if some wealthy gentleman would send them the odd £260 as a Christmas-box. But as that is almost too good a thing to be hoped for, a baker's dozen of the inhabitants might make up their minds to send £20 each to the treasurer on Christmas Day, and that would be the exact money. Lest even that should also fail, we would advise 52 of the liberal-minded citizens to send £5 each; and then the £2,000 would be completed. Of course, the best plan of all would be that the rich man should send his £260, the baker's dozen their £20 each, and every one of the 52 his £5. That would be to make the endowment absolutely safe; and the additional £500 would probably justify the committee in adding a couple of children's cots to their wards. We venture, with much warmth, to commend our scheme to the consideration of the kindest hearts in Bideford.

